# Bioprospecting of Goat Rumen Microbiota for Optimum Cellulase Enzyme Production to Support Sustainable Bioenergy Systems

**DOI:** 10.3390/microorganisms13092170

**Published:** 2025-09-17

**Authors:** Kgodiso J. Rabapane, Tonderayi S. Matambo

**Affiliations:** 1 Centre of Competence in Environmental Biotechnology, Department of Environmental Science, University of South Africa’s College of Agriculture and Environmental Science, Cnr Pioneer and Christian De Wet Roads, Private Bag X6, Florida 1710, South Africa; kgodiso93@gmail.com; 2 Institute for Catalysis and Energy Solutions, University of South Africa’s College of Science, Engineering, and Technology, Cnr Pioneer and Christian De Wet Roads, Private Bag X6, Florida 1710, South Africa; 3 CAES Bioenergy Consortium, University of South Africa’s College of Agriculture and Environmental Science, Cnr Pioneer and Christian De Wet Roads, Private Bag X6, Florida 1710, South Africa

**Keywords:** pretreatment, lignocellulosic biomass, bioenergy, cellulase

## Abstract

This study reports the isolation and optimization of cellulase-producing bacteria from the gastrointestinal tract of South African goats for the pretreatment of lignocellulosic biomass in bioenergy applications. Among the isolates, three strains, *Bacillus* KC50, *Bacillus* KC70, and *Proteus mirabilis* KC94, were identified by 16S rDNA sequencing. To our knowledge, this is the first report of cellulolytic optimization in *P. mirabilis* derived from goat rumen. Enzyme production was optimized under varying pH, temperature, and incubation conditions. *P. mirabilis* KC94 exhibited robust enzyme activity at pH 7 and 35 °C, with stability across a broader range than the *Bacillus* strains. Peak activity occurred at 84 h of incubation, reflecting strain-specific metabolic adaptation. The presence of organic solvents and surfactants inhibited enzyme activity, whereas mild oxidative stress induced by H_2_O_2_ stimulated cellulase production. Amplification of GH39, GH45, and GH48 genes revealed KC94’s strong genetic potential for efficient lignocellulose degradation. These findings highlight the biotechnological potential of rumen-derived cellulolytic bacteria, particularly *P. mirabilis* KC94, for advancing sustainable bioenergy systems.

## 1. Introduction

Lignocellulosic biomass presents an abundant and renewable resource for biofuel and biochemical production [1,2]. Effective enzymatic hydrolysis of cellulose and hemicellulose is essential to unlock the fermentable sugars within these complex substrates [3,4,5,6]. Therefore, utilizing cellulase-producing bacteria offers an efficient and environmentally friendly approach to lignocellulosic degradation, paving the way for enhanced biofuel and value-added chemical production [7].

Among cellulase-producing bacteria, members of the *Bacillus* genus, such as *Bacillus subtilis* [8,9,10], *Bacillus amyloliquefaciens* [11], and *Bacillus cereus* [12], have demonstrated significant cellulolytic activity due to their robust enzyme production and adaptability to various environmental conditions, as well as rapid growth rates. In contrast, *P*. *mirabilis* is rarely associated with cellulose-degrading or cellulolytic activity. While limited studies have described weak to moderate degradation of polysaccharide substrates such as cellulose, dextran sulfate, and mannose-based glycopolymers [13,14], only one study has demonstrated extracellular β-glucosidase production in *P. mirabilis* VIT117, isolated from shellfish waste. In that study, enzyme activity was maximized at pH 7.0 and ~72 h of incubation, with stable activity under mesophilic conditions (35–37 °C), highlighting its adaptability to physiological environments [15]. However, its presence in the gastrointestinal tracts of ruminants suggests a possible role in fiber degradation that remains poorly understood and warrants further investigation. Exploring such unconventional cellulolytic candidates may reveal overlooked microbial contributions and expand the diversity of biocatalysts for biomass utilization. The goat gastrointestinal tract represents a particularly underexplored microbial niche. Ruminant microbiota is well known for its role in breaking down complex plant materials, and isolating novel cellulolytic strains from this environment could offer new opportunities for optimizing lignocellulosic pretreatment [16,17,18]. Despite the global interest in microbial cellulases, there is still limited knowledge of cellulolytic diversity within African goat populations, leaving a knowledge gap in both microbial ecology and applied biotechnology.

Some of the key factors influencing microbial cellulase production include pH, temperature, incubation time, inoculum size, and the presence of surfactants and organic solvents [19]. Therefore, it is crucial to optimize these parameters in order to achieve maximum enzyme activity and efficient substrate degradation. In this study, cellulase-producing bacteria were isolated from the gastrointestinal tract of South African goats, and three promising strains were identified: *Bacillus* KC50, *Bacillus* KC70, and *Proteus mirabilis* KC94. To our knowledge, this is the first report of cellulolytic optimization of *P. mirabilis* in the goat rumen. The specific objectives were to characterize and identify cellulase-producing strains from goat gastrointestinal samples, to optimize key environmental factors (pH, temperature, and incubation time) influencing their cellulase production and activity, and to assess the effects of surfactants, solvents, and oxidative stress on enzyme performance, as well as to characterize lignocellulose-degrading genes in KC94 that underpin its potential for biomass conversion. By addressing these objectives, the study contributes to expanding the potential of cellulolytic bacteria and advancing microbial pretreatment strategies for sustainable lignocellulosic biomass utilization. This study was informed by our previous profiling of CAZyme-producing microorganisms [18], which revealed the presence of *P. mirabilis* harboring genes associated with cellulose-oligosaccharide metabolism, suggesting its potential as a previously underexplored cellulolytic candidate.

## 2. Materials and Methods

### 2.1. Ethical Statement

This study was conducted in the College of Science, Engineering, and Technology of the University of South Africa. Ethics approval was granted by the School of Engineering Ethics Review committee, chaired by Dr. Walied A. Elsaigh, and assigned reference number 2020/CSET/SOE/028. Because of the nature of the study involving isolation of microorganisms form goat GIT it was submitted for further approval within the College of Agricultural and Environmental Sciences Animal Research Ethics Committee (CAESAREC) of the University of South Africa, chaired Dr. A Wilson., where it was also granted and assigned the following reference number: 2021/CAES_AREC/097, chaired Ensuring humane treatment of the animals during slaughtering and compliance with regulations. The Chief State Veterinarian of South African Agriculture and Rural Development, Dr S Kamudyariwa, also approved this study and allocated it the reference number UNISA-01-2021-KJR.

### 2.2. Bacterial Screening, Isolation, and Qualitative Assay

Forty gut samples from different compartments (rumen, reticulum, omasum, and abomasum) of ten goats were grown in Brain Heart Infusion Broth (BHIB) (Lasec, SA, (Pty) Ltd., Cape Town, South Africa) to promote the growth of fastidious microorganisms under anaerobic conditions. The gastrointestinal samples were acquired as outlined [18]. About 1% of the samples were inoculated into 250 mL BHIB at pH 7.01 and incubated at 41 °C in a water bath for 5 days with regular shaking to mimic the goat’s gastrointestinal (GIT) environment. Anaerobic conditions were maintained using 500 mL reactors, with gas release measured using the water displacement method. After cultivation, the cultures were stored at −86 °C in 20% glycerol for long-term storage, creating a biobank. This biobank allows for future research without requiring additional goat slaughtering, promoting food security. Approximately 250 µL from the enrichment was used to inoculate 50 mL of the cellulase production medium, as listed in Supplementary Material Table S1. The pH was maintained at 7.0 and incubated at 37 °C for 48 h following serial dilution using 0.9% NaCl. A volume of 100 µL from each 10^−8^ dilution was plated and spread on 0.5% (*w*/*v*) Carboxymethyl Cellulose (CMC) (St. Louis, MO, USA) for cellulolytic activity. Plates were incubated at 37 °C for 48 h in an inverted position. Isolated cultures were purified by repeatedly picking and streaking on agar plates to obtain pure isolates. The plates were then flooded with 0.1% Congo red (*w*/*v*) solution for 30 min and then washed with 1 M NaCl for 15 min [20,21,22,23]. A light yellow color around the isolate confirmed the hydrolysis of cellulose. The zone of hydrolysis was measured using a digital caliper (mm). A negative control (non-cellulolytic strain) was used to rule out the false positives. The relative enzyme activity was calculated using Equation (1) [24,25]:

(1) $$REA=\frac{CZD}{CD}$$

where REA: relative enzyme activity, CZD: clear zone diameter, and CD: colony diameter. Positive isolates were stored in 20% glycerol stocks at −86 °C.

### 2.3. Identification of the Cellulase-Producing Bacteria by 16S rDNA Sequencing

For identification, primers 27F (5′-AGAGTTTGATCMTGGCTCAG-3′) and 1492R (5′-CGGTTACCTTGTTACGACTT-3′) [26,27] were used to amplify the 16S rDNA gene of the pure isolates using extracted genomic DNA (*Quick*-DNA Fungal/Bacterial extraction kit, Zymo Research, Irvine, CA, USA) as a template for the polymerase chain reaction (PCR). Amplified genes were visualized using 1% agarose gel electrophoresis. The PCR products of the amplified 16S rDNA genes were sequenced at Inqaba Biotech (Pty) Ltd. (Pretoria, South Africa) using the ABI Big dye V3.1 kit following the manufacturer’s protocol and sequenced using the ABI 3730xl Genetic Analyzer (Applied Biosystems, Thermo Fisher Scientific, Waltham, MA, USA) [28]. Sequences for KC40, 50, 70, and 94 were aligned using ClustalW multiple alignment on BioEdit v7.2 software [29] and MAFFT—an online version of a multiple sequence alignment program. The obtained 16S rRNA gene sequence was submitted to NCBI GenBank, and a phylogenetic tree was constructed using the neighbor-joining method (using MEGA 11.0) [30]. Bootstrap resampling analysis for 1000 replicates was performed to estimate the confidence of the tree topologies [31]. Operational taxonomic units (OTUs) were generated using MOTHUR (v1.48.0) to group similar species at a 98% similarity threshold. The representative OTU sequences were then compared to sequences in the GenBank database for identification [32].

### 2.4. Effects of pH, Temperature, and Incubation Period

The selected isolates were grown at 37 °C for 24 h. After which, they were used as a 1% inoculum to inoculate CMC production media as a carbon source at various pHs ranging from 4.0 to 9.0 (pH 4.0 and 5.0 with Acetate Buffer, pH 6.0 and 7.0 with Phosphate Buffer, pH 8.0 and 9.0 with Tris-HCl) at temperatures 35–65 °C. The cellulase activity was checked using the DNS method [33]. The pH and temperature with the highest activity were selected and analyzed further for the effect of the incubation period at 12 h intervals. To determine the cellulase activity produced by the isolate, the cultures were centrifuged at 4 °C (4400 rpm) for 15 min, and the cell-free extracts (CFEs) were assayed for cellulase activity [34]. Two hundred and fifty microliters (250 µL) of the buffered CMC (0.5%) at pH 7.0 was equilibrated for 5 min at 35 °C in a water bath. After 5 min of equilibration, 250 µL of the CFE was transferred to a test tube to start the reaction and stopped after 30 min [35]. Then, 500 µL of DNS (3.5-dinitrosalicylic acid) reagent (Glentham Life Sciences Ltd., Corsham, UK) was added to each test tube to stop the reaction. The test tubes were placed in boiling water for 5 min [36] and then cooled to room temperature with running water. Thereafter, 2 mL of distilled water was added to the test tubes, and the absorbance was measured at 540 nm. Then, the absorbance was converted to mg/mL of glucose from the calibration curve. The substrate and DNS reagent were mixed first, and the CFE was added afterward for the blank reaction [36]. This experiment was performed in triplicate, and the means ± standard deviation were used to plot the figures. One unit of cellulase activity was defined as the amount of enzyme that liberated 1 µmol of glucose equivalents under the assay conditions. The formula below (Equation (2)) was used to calculate the enzyme activity [35,37,38].

(2) $$\frac{U}{ml}=\frac{\left[glucose\frac{mg}{ml}\right]}{Time\left(min\right)\times VS\;\left(ml\right)\times MW\;glucose\frac{mg}{mmol}\;}\times 1000$$

where VS = Volume of Substrate and MW = Molecular Weight.

### 2.5. Effects of Organic Solvents and Surfactants on Cellulase Production

To investigate the effects of organic solvents and surfactants on the isolate’s ability to produce cellulase, the production medium was supplemented with a low concentration (0.5%) and a high concentration (2%) of various organic solvents and surfactants under optimum conditions. We tested two solvent/surfactant/oxidizing levels, 0.5% (low, process-relevant) and 2% (high, stress-level), deliberately to capture both typical operational exposure and an upper bound for enzyme tolerance. While many previous studies use 0.1–0.2% for low concentrations and ~1% for higher levels [21,39]. These are often specific to conventional pretreatment of industrial waste. In contrast, we included 0.5% as a more realistic low-level condition relevant to some detergent-assisted pretreatment streams, and 2% as an extreme stress condition to explore enzyme stability at the upper range of reported or conceivable industrial surfactant exposure. This approach allows us to evaluate both practical applicability and enzyme robustness under more challenging conditions.

### 2.6. PCR Amplification of Possible Cellulase and Hemi-Cellulase Gene Fragments on KC94

The Touchdown (TD) PCR settings on Supplementary Table S3 were used to amplify the targeted fragments using the T100 Thermal Cycler (Bio-Rad, Hercules, CA, USA). The DNA template concentration was 9.04 ng/µL as determined by the Qubit 3^®^ (Applied Biosystems, Thermo Fisher Scientific, Waltham, MA, USA) high-sensitivity assay. OneTaq 2X Master Mix (New England Biolabs, Ipswich, MA, USA) was used for amplification of GH45 and GH48, while Q5 High-Fidelity 2X Master Mix (NEB) was used for GH39. The targeted genes and their corresponding enzymes were as follows: GH39 (PF01229), β-xylosidase; GH45 (PF02015), β-1,4-endoglucanase; and GH48 (PF02011), cellobiohydrolase. The list of degenerate primers used in this study is also included in the Supplementary Materials Table S3, as adopted from [40]. KC94 was selected for PCR-based gene amplification and enzyme-specific validation. This decision was based on preliminary optimization experiments, which consistently identified KC94 as the most robust cellulolytic strain. Therefore, KC94 was considered the most representative candidate for further molecular characterization.

### 2.7. Statistical Analysis

Each experiment was performed in triplicate (*n* = 3) to accurately capture variability within the dataset, and the standard deviation for each experimental result was calculated and populated using Microsoft^®^ Excel^®^ for Microsoft 365 MSO (Version 2508 Build 16.0.19127.20192). Statistical analysis was performed using two-way ANOVA with Tukey’s post hoc test (*p* < 0.05) using IBM SPSS Statistics (version 30.0.0.0).

## 3. Results

### 3.1. Qualitative Assay of Cellulase-Producing Bacteria

Twelve (12) bacterial isolates were isolated from the ruminal fluid samples, of which 4 isolates (33%) showed clear zone hydrolysis on CMC agar upon primary screening. About (4) bacterial isolates exhibited good cellulase activity, confirmed by sub-culturing on 1% CMC for 72 h at 37 °C, flooding the plates with 0.1% Congo red, and washing off with 1 M NaCl. The bacterial isolates exhibited a significant clear zone diameter and relative enzyme activity (REA) ranging from 16.21 mm to 78.62 mm and between 1.26 and 4.15, respectively (Table 1). A univariate analysis of variance (GLM) was conducted to assess differences in REA among isolates. The analysis revealed a significant effect of isolate on REA (F_3_,_8_ = 5480.94, *p* < 0.001, partial η^2^ = 1.000), indicating that REA differed substantially between isolates. Levene’s test confirmed homogeneity of variance (*p* = 0.143), satisfying the assumption for ANOVA. Post hoc comparisons using Tukey’s HSD indicated that all pairwise differences between isolates were statistically significant at the 0.05 level (*p* < 0.001 for all comparisons). Specifically, KC94, which exhibited the highest REA, was significantly greater than KC70, KC40, and KC50, while KC50 had the lowest REA.

Correlation analysis revealed strong relationships among the measured variables. Colony diameter was highly positively correlated with clear zone diameter (r = 0.990, *p* < 0.001), and both colony diameter. Out of the 4 isolates subjected to secondary screening, 3 showed significant cellulase activity by producing relatively high amounts of glucose when assayed at physiological conditions (37 °C and pH 7).

### 3.2. Bacterial Identification

Identification through the 16S rDNA sequence revealed KC40 to be closely related to *Bacillus licheniformis*, KC50, KC70 with *Bacillus subtilis*, and KC94 with *P. mirabilis*.

**Figure 1:**
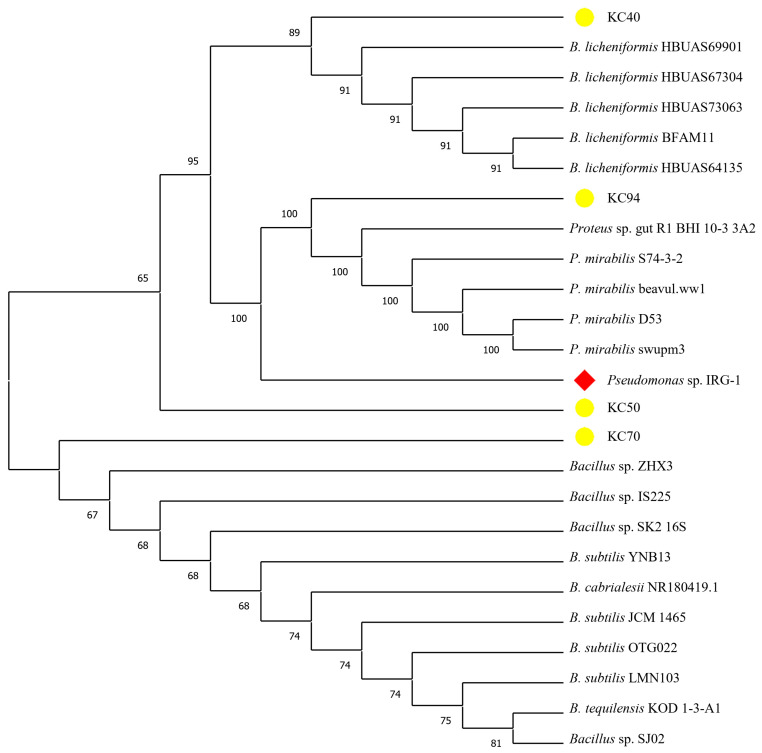
Phylogenetic tree showing the relationship between isolate KC40, KC50, KC70, and KC94 (highlighted in yellow). The phylogenetic analysis used the neighbor-joining method, and the significance of junctions was established using the bootstrap method (1000 replicates). *Pseudomonas* sp. *IRG-1*, highlighted in red, was used as an outlier.

*Pseudomonas* sp. IRG-1 was used as an outlier to root the tree, representing a reference point for comparison. Based on the phylogenetic findings, isolate KC40 was identified as *Bacillus licheniformis.* Meanwhile, KC50 and KC70 were classified within the *Bacillus* genus but may represent different subspecies or closely related species, warranting further investigation. KC94 was identified as *P. mirabilis* (Figure 1). Bootstrap analysis provided additional confidence, with high values (100) observed in the clusters involving *P. mirabilis* and among the *Bacillus* sp., supporting the robustness of these relationships. The inclusion of *Pseudomonas* sp. *IRG-1* as an outgroup confirmed that the isolates are more closely related to each other than the outlier. This separation validated the tree’s internal structure and highlighted the distinct evolutionary pathways of the *Bacillus* and *Proteus* sp.

### 3.3. Optimization Studies

#### 3.3.1. The Effect of pH

**Figure 2:**
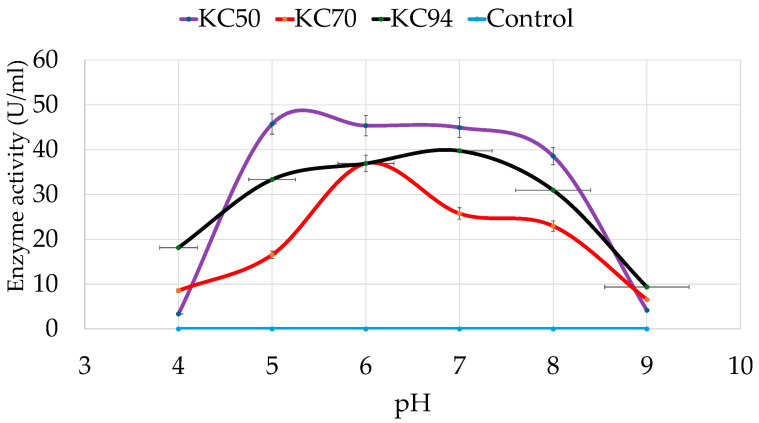
pH-dependent growth and enzyme activity profiles of the isolated strains. Error bars represent mean ± SD of three independent replicates (*n* = 3). Mean standard deviation for all the values is <±5.0%. Statistical analysis was performed using one-way ANOVA with Tukey’s post hoc test (*p* < 0.05). All pairwise differences among pH levels and strains were statistically significant (Tukey’s HSD, *p* < 0.05).

The effects of pH on the bacterial strains’ cell growth and cellulase production were assessed with CMC after growing in various pH levels ranging from 4 to 9 for 24 h. As shown in Figure 2, KC50, KC70, and KC94 have optimum pHs of 5, 6, and 7, respectively. KC50 strain exhibited a significant increase in cellulase activity at pH 5 and maintained a relatively stable activity level from pH 5 to pH 7, with a slight decrease at pH 8. However, under extreme acidic (pH 4) and alkaline (pH 9) conditions, KC50 exhibited negligible cellulase activity, indicating a narrow optimal pH range.

KC70, on the other hand, showed a more consistent but lower cellulase activity across the pH spectrum. At pH 5 and 6, KC70 demonstrated relatively good activity, but it dropped significantly as the pH approached both extremes, with a notable decrease at pH 8 and minimal activity at pH 9. KC94 exhibited significant growth over a wide pH range, reaching its optimum at pH 7 while retaining considerable activity between pH 5 and 8. A similar optimum pH strain was also observed [35], and a significant drop was observed at pH 8. At pH 9, KC94 also showed higher average enzyme activity than the other isolates, although the variability between replicates was larger, as indicated by the error bar.

#### 3.3.2. The Effects of Temperature

**Figure 3:**
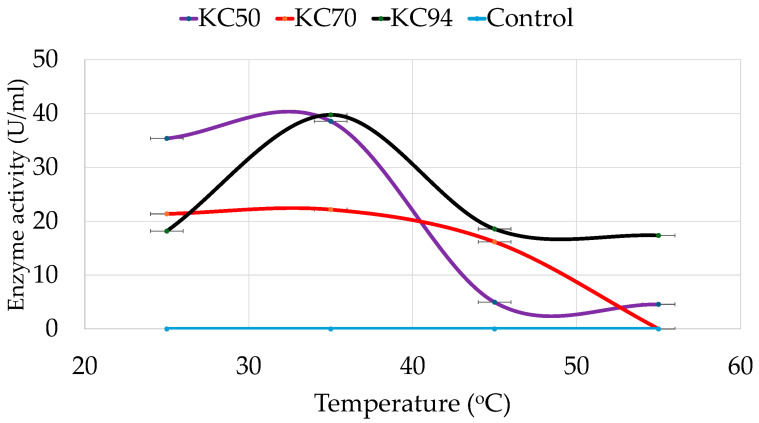
Effect of temperature on growth and cellulase production of KC50, KC70, and KC94. Error bars represent mean ± SD of three independent replicates (*n* = 3). Statistical analysis was performed using one-way ANOVA with Tukey’s post hoc test (*p* < 0.05). All pairwise differences among temperature levels and strains were statistically significant (Tukey’s HSD, *p* < 0.05).

The effect of temperature on cellulase production was also determined for all the strains, revealing distinct thermal tolerance patterns. Accordingly, KC50 exhibited optimal cellulase activity at 35 °C, indicating its mesophilic nature. At 25 °C, KC50 retained significant activity, but enzyme efficiency declined sharply at 45 °C and 55 °C (Figure 3). On the other hand, KC70 demonstrated lower enzyme activity than KC50 across all temperatures, with peak cellulase production at 35 °C. A steady decline was observed as the temperature increased, and no detectable activity was recorded at 55 °C.

Meanwhile, KC94 exhibited the highest temperature tolerance among the tested strains, maintaining substantial activity from 25 °C to 55 °C. Like KC50 and KC70, KC94 displayed peak activity at 35 °C but retained significant cellulase function at both 45 °C and 55 °C. Interestingly, although KC70’s activity was less throughout the thermals levels, it retained noticeable activity at 45 °C compared to KC50.

#### 3.3.3. The Effects of the Incubation Period

Interest has been placed on cellulase-producing bacteria due to their ability to grow at a rapid rate. Various studies have reported high enzyme production at 24 [41], and 48 h [35]. Meanwhile, others have reported 72 h [15,35,37,42], and as long as 5–7 days [30]. In a study by Gaur and Tiwari, enzyme production was observed at 12 h, with maximum production observed at 48 h when investigated on various agro-wastes [21]. In this study, cellulase production was monitored over time up to 84 h. As a result, KC50 displays consistent performance and sustained productivity over time, marked by an impressive early growth phase characterized by a sharp increase at the beginning. It quickly rises to about 50 units after 36 h, indicating that KC50 has a fast initial growth rate due to either rapid cellulase production or expansion. However, its growth reaches a peak of around 50 units and then gradually declines [43]. Meanwhile, KC70 shows a slower but steady increase, reaching around 30 units. Its growth rate is less steep than that of KC50, suggesting a prolonged but less intense period of activity.

**Figure 4:**
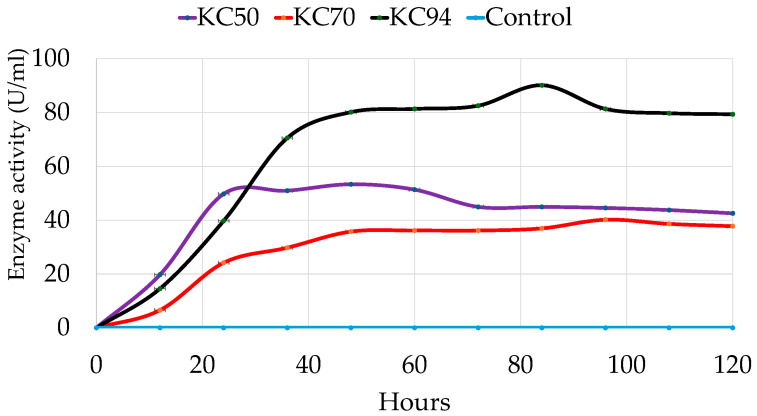
Determination of optimal incubation time for maximal cellulase activity in all tested isolates. Error bars represent mean ± SD of three independent replicates (*n* = 3). Statistical analysis was performed using two-way ANOVA with Tukey’s post hoc test (*p* < 0.05). All pairwise differences among time points and trains were statistically significant (Tukey’s HSD, *p* < 0.05).

Interestingly, KC94 demonstrates the best performance, peaking at approximately 90 units at 84 h and consistently maintaining a high level (Figure 4). This suggests that KC94 is highly effective throughout the entire duration, likely indicating strong cellulase activity or steady biomass production [44]. A two-way ANOVA revealed significant main effects of incubation time and strain on activity, as well as a significant incubation time and strain interaction, indicating that strain effects varied across time points. Post hoc tests showed that KC94 consistently yielded the highest values, followed by KC50 and KC70, with significant differences across all incubation time points.

### 3.4. The Effects of Organic Solvents, Surfactants, and Oxidizing Agent on Cellulase Production

#### 3.4.1. Solvents

**Figure 5:**
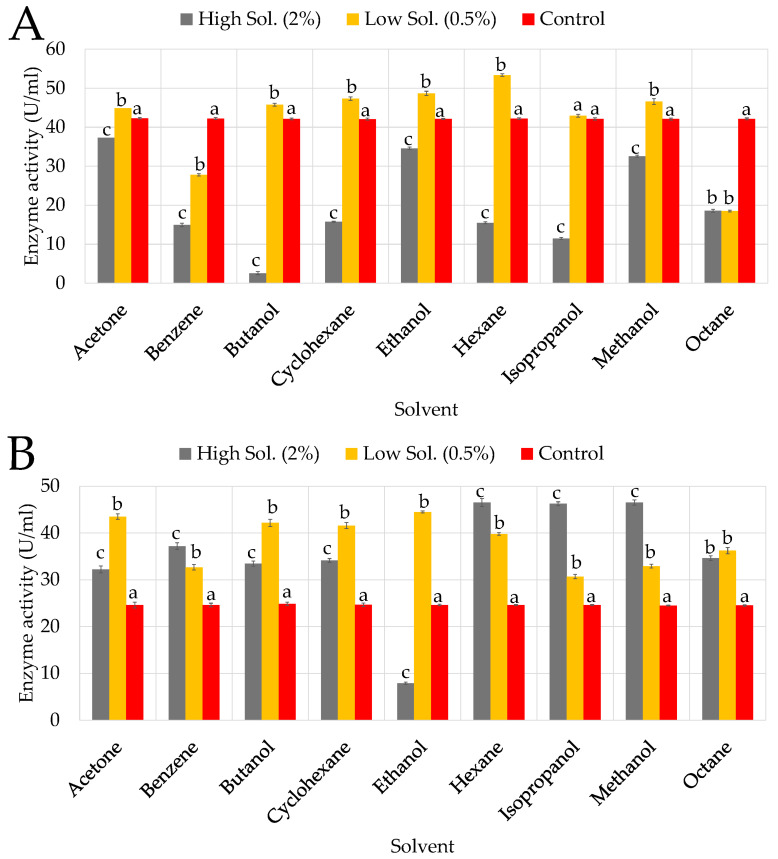

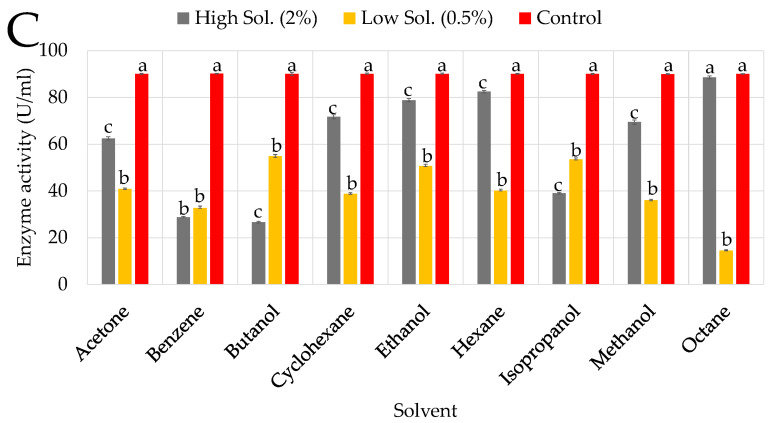
Influence of organic solvents on growth and enzyme activity of the three isolates: KC50 (**A**), KC70 (**B**), KC94 (**C**). Error bars represent mean ± SD of three independent replicates (*n* = 3). Statistical analysis was performed using two-way ANOVA with Tukey’s post hoc test (*p* < 0.05). Letters above bars indicate statistical differences within each solvent: bars labeled “a” are the control (baseline), “b” = significantly different from control, “c” = significantly different from both control and low (0.5%) concentration, and bars sharing the same letter are not significantly different from each other (Tukey’s HSD post hoc test, *p* < 0.05).

KC50’s ability to produce cellulase was significantly inhibited in the presence of butanol, isopropanol, and benzene, with residual activities (RA) of 2.55%, 11.36%, and 14.95%, respectively (Figure 5A). Cyclohexane, hexane, and octane exhibited moderate inhibition, with RAs of 15.75%, 15.35%, and 18.56%, respectively, indicating that KC50 is less tolerant to non-polar solvents. Moderate tolerance was observed in the presence of ethanol (34.56%) and acetone (37.36%), suggesting that KC50 retains some activity in the presence of these less toxic solvents. KC70 generally exhibited moderate tolerance to organic solvents but was particularly sensitive to ethanol at higher concentrations (Figure 5B), which strongly inhibited cellulase production. Non-polar solvents such as hexane and octane caused moderate inhibition but, in some cases, even outperformed the control, suggesting KC70 maintains enzyme structure stability in hydrophobic solvents.

At a higher concentration (2%), butanol significantly inhibited KC94’s cellulase production (Figure 5C), resulting in approximately 26.55% RA, likely due to its toxic effects, causing membrane disruption and enzyme inactivation [45,46]. Isopropanol exhibited similar inhibition (38.96% RA), and benzene also had a notable inhibitory effect (28.96% RA). Moderate inhibition was observed with acetone (62.56% RA), reflecting its intermediate polarity [47]. Despite these effects, KC94 showed notable tolerance to ethanol (78.96% RA), likely due to ethanol’s stabilizing effects on the enzyme’s hydrophobic core [48]. Likewise, KC94 maintained significant cellulase production in cyclohexane (71.76% RA), hexane (82.56% RA), and octane (88.57% RA).

#### 3.4.2. Surfactants and Oxidizing Agent

**Figure 6:**
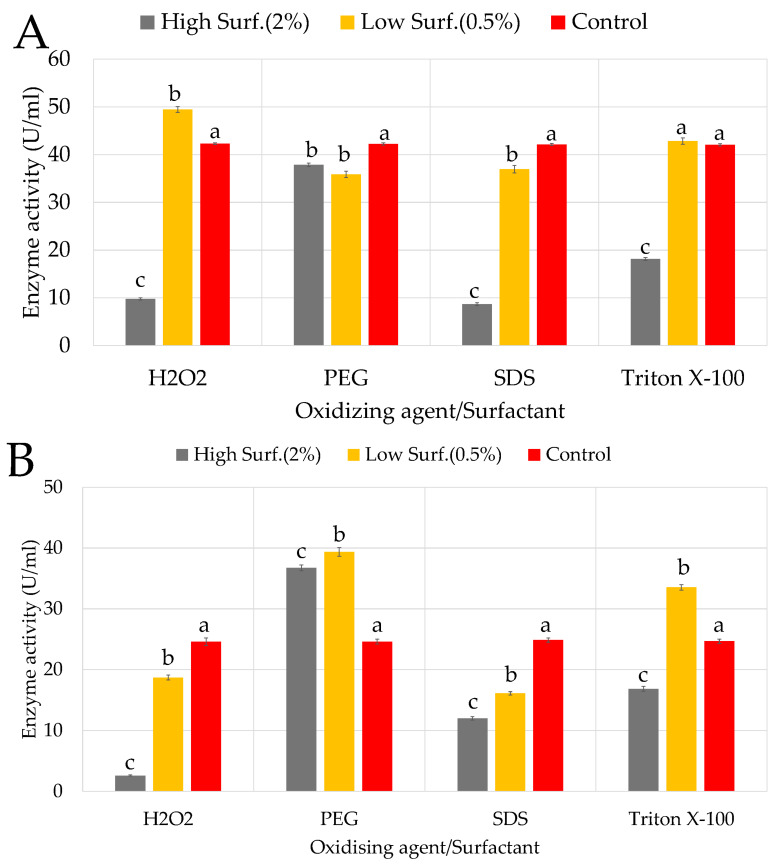

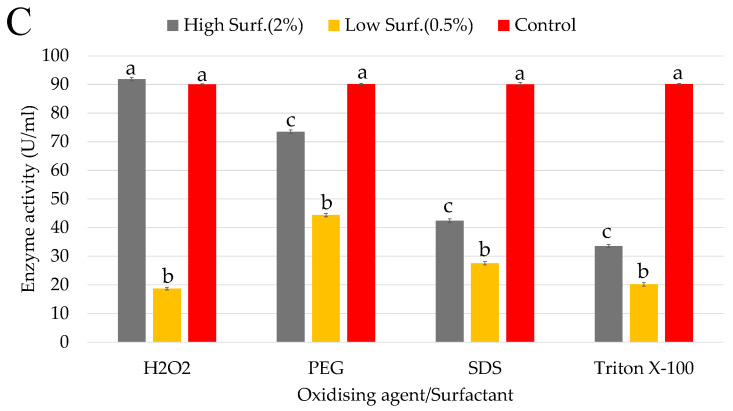
Impact of selected surfactants and oxidizing agents on enzyme activity profiles of the three isolates: KC50 (**A**), KC70 (**B**), and KC94 (**C**). Error bars represent mean ± SD of three independent replicates (*n* = 3). Statistical analysis was performed using two-way ANOVA with Tukey’s post hoc test (*p* < 0.05). Letters above bars indicate statistical differences within each surfactant/oxidizing agent: bars labeled “a” are the control (baseline), “b” = significantly different from control, “c” = significantly different from both control and low (0.5%) concentration, and bars sharing the same letter are not significantly different from each other (Tukey’s HSD post hoc test, *p* < 0.05).

The effects of surfactants at high concentrations showed strong inhibition by SDS and Triton X-100 on KC50 (Figure 6A), with only 8.56% and 18.16% RA, respectively. Triton X-100, a non-ionic surfactant, likely disrupted the enzyme’s hydrophobic regions, leading to destabilization and reduced activity. PEG exhibited moderate inhibition with 37.75% RA, likely due to its interference with enzyme stability through hydration layer modification. Hydrogen peroxide (H_2_O_2_) as an oxidizing agent appeared particularly unfavorable for KC50 (9.75% RA), though cellulase activity was slightly higher at lower concentrations (0.5%). Notably, H_2_O_2_ stimulated cellulase production (49.36% RA), suggesting that mild oxidative stress might activate protective pathways.

KC70 exhibited varied responses to different surfactants, with enzyme production significantly influenced by both type and concentration (Figure 6B). High concentrations (2%) of SDS and H_2_O_2_ strongly inhibited cellulase production, while lower concentrations (0.5%) of PEG and Triton X-100 stimulated enzyme activity. Regarding surfactants, SDS and Triton X-100 strongly inhibited cellulase activity on KC94, with RAs of 19.56% and 22.76%, respectively (Figure 6C). These surfactants likely disrupted enzyme conformation by interfering with hydrophobic regions, reducing functionality. PEG exhibited moderate inhibition (47.65% RA), while H_2_O_2_ had a relatively mild effect (54.36% RA), suggesting that KC94 possesses mechanisms to tolerate oxidative stress. At lower concentrations (0.5%), PEG, SDS, and Triton X-100 had less inhibitory effects, while H_2_O_2_ slightly stimulated cellulase production (64.36% RA), possibly due to the activation of oxidative stress response pathways.

### 3.5. Diversity of Possible Cellulase and Hemi-Cellulase Gene Fragments in KC94

**Figure 7:**
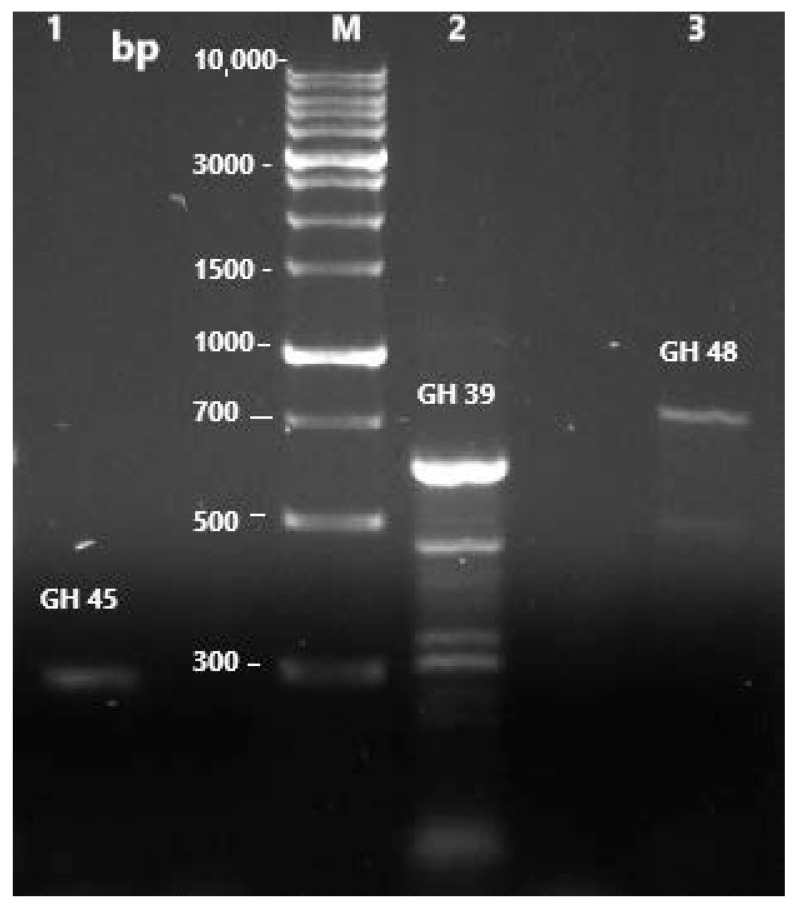
1% agarose gel electrophoresis of the Touchdown PCRs. M: 1 kb DNA marker; 1: GH45 coding for β-1,4-endoglucanse (377–412 bp), 2: GH39 coding for β-xylosdase (223–230 bp), 3: GH48 coding for cellobio-hydrolase (420 bp). The empty lane between lanes 2 and 3 was the control containing all the PCR reagents except the DNA fragment.

The GH45 family gene coding for β-1,4-endoglucanase was successfully amplified at the expected size range of 377–412 bp (Figure 7). Similarly, amplification of the GH39 family, which codes for β-xylosidase, produced clear products at the expected size of 223–230 bp. Interestingly, additional bands were also observed from the GH39 amplification, with sizes ranging from 321 to 665 bp. For GH48, two distinct amplicons were obtained, with sizes between ~480–702 bp.

## 4. Discussion

### 4.1. Cellulase Activity Identified by Qualitative Assay 

The qualitative CMC plate assay confirmed that the hydrolysis zone size correlates with cellulase production capacity, consistent with earlier reports [49]. Moderate cellulase activity is exhibited by KC40, as indicated by the CZD, which is over twice the size of the colony diameter. The REA of 2.21 suggests it is a decent cellulase producer but less efficient than the other strains, like KC94 and KC70. Thus, the moderate CZ indicates that KC40 can degrade cellulose but not as aggressively as the other strains. Interestingly, the largest CD was observed in KC50, which suggests rapid growth because its CZ is slightly larger than the CD, resulting in a lower REA of 1.26. Therefore, although KC50 grows fast, its ability to produce cellulase may not be attributed to its growth rate, meaning that it might not produce significantly high levels of cellulase as compared to the other strains. Meanwhile, KC70 exhibits a CZ that is almost three times larger than its CD, indicating a strong cellulase activity relative to its size. The REA of 2.66 is substantially higher and indicative of a potent cellulase producer [24]. Although KC70 is more efficient in cellulase production than KC40 and KC50, it still falls short compared to KC94, which has the highest REA (4.15), indicating the highest production relative to its colony size. The CZ of KC94 is approximately four times larger than the CD, suggesting its ability to break down cellulose efficiently. Although its colony size is smaller, it produces the highest degradation activity, making it the most efficient cellulase producer among all the strains. This pattern highlights the importance of considering relative activity measures rather than absolute colony or zone size when comparing isolates [49]. The variation in CZD and REA could be attributed to the preference for pH. The media for the primary screening was at 7, favoring the KC94 strain based on the pH assay conducted in the subsequent sections, and may have impacted the growth of isolates KC50 and KC70, which thrived at pH 5 and 6, respectively.

### 4.2. The Effects of pH, Temperature, and Incubation Period

#### 4.2.1. pH

The differences in pH response among isolates highlight their potential ecological roles and industrial relevance. KC50 showed a narrow pH optimum centered at 5–7, which aligns with earlier reports of *Bacillus subtilis* cellulases exhibiting optimal activity at slightly acidic to neutral conditions [50]. Given that the rumen environment is generally maintained within a regulated pH range of 6.2–7.0 [51]. KC50’s stable activity within this range indicates that the rumen environment is conducive for it to produce cellulase. Its efficiency under near-neutral pH conditions suggests that KC50 could contribute substantially to cellulose degradation under typical rumen conditions, particularly when compared to strains that lose activity at neutral pH. This supports the potential relevance of KC50 as an effective cellulase producer within the rumen ecosystem, where maintaining fiber digestion efficiency is crucial for host nutrition.

Meanwhile, KC70 exhibited moderate and more evenly distributed activity, which is somewhat more resilient to changes in pH than KC50 but has a less efficient cellulase production capacity overall. The moderate performance of KC70 could be attributed to its ability to maintain some enzyme functionality under diverse conditions, though it still experiences a decrease at higher pH. Although KC70 produces less cellulase than KC50 under optimal conditions, it could still contribute to cellulose degradation in the rumen environment (6.2–7.0) because its activity is sustained within the physiological pH of the rumen. This observation supports the concept that *Bacillus* species, including KC70, can maintain some cellulase activity in a broader pH range. However, efficiency decreases as the pH moves away from its optimal conditions [50]. This is a common observation in microorganisms found in neutral or slightly acidic environments [35,43].

KC94 demonstrated both high activity and broad pH tolerance, with optimum cellulase activity at pH 7 and retention across pH 5–8. This broad range of pH tolerance suggests that KC94 might possess efficient mechanisms for regulating pH, such as effective ion exchange systems that protect cellular components from pH-induced stresses [52]. These properties make KC94 an ideal option for applications such as lignocellulosic biomass pretreatment, where pH levels often fluctuate as the substrate is gradually broken down. This finding aligns with previous studies reporting that *P. mirabilis* VIT117 can tolerate a broad pH range, suggesting the presence of mechanisms that help maintain cellular stability under varying pH conditions [15]. Meanwhile, urease activity has been identified as a common pH-regulating mechanism in *P. mirabilis* [53,54]. The current study did not assess urease production. Therefore, other regulatory pathways, such as ion exchange systems or membrane adaptations, may contribute to KC94’s ability to function efficiently across different pH levels.

Unlike KC50, which performs best in a narrow pH range, and KC70, which shows lower overall activity, KC94 combines both high activity and tolerance across a broader pH spectrum. A two-way ANOVA revealed that pH significantly affected activity (*p* < 0.001), which also differed among strains. There was a significant (*p* < 0.001) pH and strain interaction, indicating that the influence of strain activity depended on the pH level. Post hoc comparisons showed that KC94 and KC50 generally exhibited the highest activity, followed by KC70. Activity varied significantly across pH levels, with maximal values observed around pH 6–7 and minimal activity at extreme pH values.

In the rumen, pH fluctuates with diet, often ranging from 5.5 to 5.8 [55,56,57] when fed a high-grain diet, and to approximately 7.0 when fed forage-rich diets. Many fibrolytic bacteria, such as *Fibrobacter succinogenes* and *Ruminococcus flavefaciens*, are highly sensitive to pH drops, losing cellulase activity below 6.0 [58]. KC94’s acid tolerance contrasts with these typical rumen cellulolytics, suggesting it could persist and remain active in suboptimal ruminal pH conditions, such as during sub-acute ruminal acidosis (SARA). This trait could provide ecological resilience and industrial utilization in acidic pretreatment environments.

#### 4.2.2. Temperature

The three strains exhibited distinct thermal tolerance patterns. KC50, peaking at 35 °C, demonstrates a mesophilic profile similar to *Bacillus subtilis* cellulases, which also lose stability at elevated temperatures [50]. Although its peak activity is slightly below the rumen temperature (38–42 °C) [59,60]. KC50 still retains significant activity at moderate temperatures. This suggests that in the rumen environment, KC50 could maintain functional cellulase activity, but may not perform at maximum efficiency due to its reduced stability at higher temperatures. This further suggests that KC50 is well adapted to moderate temperatures but lacks the thermostability required for high-temperature industrial processes. KC70 showed reduced overall activity and a sharp loss at 55 °C, indicating weak thermal adaptation. This suggests that KC70 possesses limited thermal adaptation, making it less suitable for applications requiring high-temperature tolerance. The strain’s limited thermal tolerance makes it less adaptable to the rumen’s constant temperature of 38–42 °C, potentially restricting its contribution to fiber degradation efficiency in situ. In contrast, KC94 combined high activity with broad tolerance, maintaining substantial cellulase function even at 55 °C. These findings were also supported by the two-way ANOVA analysis, which revealed significant main effects of temperature and strain interaction, indicating that the effect of strain depended on temperature. Post hoc tests showed that KC94 and KC50 generally yielded the highest values, followed by KC70 and Control. Pairwise comparisons between temperatures revealed significant differences across all strains. This suggests that KC94 may possess mechanisms that enhance enzyme stability at elevated temperatures, such as increased hydrogen bonding or enhanced protein folding [61,62,63]. Most ruminal species display temperature optima close to 38 °C and decline sharply from 43 °C [64]. By contrast, KC94 maintains activity above 45 °C, which is unusual for rumen isolates, suggesting an advantage for consistent cellulose degradation in the rumen while also making it suitable for industrial processes that require higher operational temperatures. To our knowledge, no prior study has reported thermostable cellulases from *P*. *mirabilis* of rumen origin. These findings align with previous research on *P*. *mirabilis* isolated from treatment sludge of textile industry factories, where laccase enzyme stability was observed over a broader temperature range (17–47 °C) due to adaptive stress responses [65]. The ability of KC94 to produce cellulases at higher temperatures suggests potential applications in biofuel production and industrial biomass degradation, where thermostability is a desirable trait.

#### 4.2.3. Incubation Period

The three isolates differed in their growth and enzyme production dynamics. KC94’s sustained high activity indicates efficient and prolonged cellulase production, making it the most reliable strain for continuous cellulose degradation [44]. KC50 displayed rapid early growth, which may be attributed to heightened metabolic activity in the early stages. The leveling off and subsequent decline could imply constraints such as a lack of nutrients, excess substrate, or the accumulation of inhibitory substances, a limitation on enzyme production, or a stress response following the initial rapid expansion [30]. Visible colonies that expand rapidly on plates can be observed (Supplementary Materials, Figure S1), confirming the fast growth noted on the plates. Hence, the high initial activity may also lead to rapid substrate exhaustion, restricting further growth or cellulase production. KC70 produced cellulase consistently, although at a reduced efficiency compared to KC50 and KC94. Rumen studies frequently report that fibrolytic bacteria exhibit distinct growth kinetics: *Fibrobacter succinogenes* rapidly colonizes fibers but declines as soluble sugars accumulate, while *Ruminococcus albus* maintains prolonged activity [66]. Therefore, KC50 is similar to *Fibrobacter*, while KC94 resembles *Ruminococcus*-like persistence. By demonstrating prolonged stability in cellulase production, KC94 may represent an untapped “slow but steady” cellulolytic niche within the rumen microbiome, complementing faster-growing degraders like KC50. Such kinetics may be advantageous in maintaining baseline cellulase activity over extended periods, though with lower efficiency than KC94, which demonstrated the most favorable profile, combining high and sustained enzyme production, which underscores its potential suitability for industrial processes and rumen biomass degradation.

### 4.3. Response of Cellulase Production to Organic Solvents, Surfactants, and Oxidizing Agent

#### 4.3.1. Organic Solvents

A two-way ANOVA was performed for each strain to assess the effects of solvent and concentration on activity. In all cases, there were significant main effects of solvent and concentration, as well as significant solvent and concentration interactions (all *p* < 0.001), demonstrating that solvent effects were strongly dependent on concentration. Post hoc Tukey HSD tests further revealed that the ranking of solvents varied somewhat among strains. For instance, KC94 exhibited superior stability, particularly in non-polar solvents. Similar observations have been reported in *Bacillus amyloliquefaciens* AK9, where cellulase showed remarkable solvent stability, with some solvents even enhancing activity [28]. A study on *Bacillus vallismortis* RG-07 demonstrated similar solvent tolerance, with cellulase activity remaining stable even at high organic solvent concentrations [21]. It is reported in the literature that non-polar solvents likely interact favorably with hydrophobic protein regions, preventing aggregation and denaturation [21,47,67]. In a similar study, the cellulases from *Promicromonospora* sp. *VP111* exhibited significant tolerance to organic solvents, particularly non-polar solvents. CMCase showed high stability and even apparent stimulation in the presence of solvents like *n*-hexane and isooctane, while FPase and β-glucosidase retained 77–95% and 79–89% residual activities, respectively. However, cellulase activity was significantly reduced in polar organic solvents, likely due to enzyme destabilization caused by solvent interactions with the enzyme’s hydration layer. Also, like the previous studies mentioned above, it suggests that non-polar solvents might enhance enzyme conformation, preventing denaturation, whereas polar solvents can wash away essential water molecules needed for enzymatic activity [68]. It is important to note that the rumen microbiome operates in a strictly anaerobic, plant fiber-rich environment and is not naturally exposed to industrial solvents used in pretreatment, such as butanol or benzene; however, microbial fermentation generates short-chain alcohols. These environments are dominated by fermentative bacteria, archaea, protozoa, and fungi specialized for cellulose breakdown, not solvent tolerance [69,70]. Therefore, the solvent resilience observed in KC94 indicates an extraordinary capacity that may be valuable for industrial applications where such solvents are present. Reports on rumen cellulases rarely test solvent effects, making this study one of the first to evaluate such tolerance in *Proteus mirabilis* from rumen origin. Furthermore, KC94’s high tolerance to ethanol (78.9% RA) is remarkable and indicates potential for bioethanol-integrated lignocellulose degradation systems, where solvent accumulation often inhibits enzyme function.

#### 4.3.2. Surfactants and Oxidizing Agent

A two-way ANOVA was also conducted to assess the effects of surfactants (PEG, SDS, Triton X-100), an oxidizing agent (H_2_O_2_), and concentration on activity. Across the three strains, there were significant main effects of both factors and their interaction (all *p* < 0.001, η^2^ > 0.99), confirming that the impact of concentration depended strongly on the surfactant and oxidizing type. Post hoc comparisons revealed consistent strain-specific trends. For instance, KC50 and KC70 showed similar trends, with high concentrations of SDS and Triton X-100 strongly inhibiting cellulase production. However, lower concentrations of PEG and oxidizing agent H_2_O_2_ resulted in mild effects or slight stimulation in both strains. KC94 showed the strongest tolerance to surfactants overall, with mild inhibition from PEG and a slight enhancement of activity with H_2_O_2_ exposure. Ionic surfactants (SDS, Triton X-100) disrupted enzyme hydrophobic interactions, leading to denaturation, whereas non-ionic surfactants (PEG, Tween) occasionally stabilized enzymes and enhanced activity, consistent with prior reports [46]. Studies have also indicated that surfactants can protect cellulases by reducing non-specific binding and enhancing enzyme stability during hydrolysis. In a study by Thomas and colleagues, Tween-80 slightly enhanced CMCase production, whereas Triton X-100, sodium lauryl sulfate (SLS), and sodium *N*-lauryl sarcosine completely inhibited cellulase activity [68]. A similar study on *Bacillus subtilis* SU40 isolated from agricultural soil also examined the effects of surfactants on enzyme activity, showing relatively stable enzyme production across different treatments. It was reported that Tween 20 resulted in the highest activity, while phenylmethylsulfonyl fluoride (PMSF) caused the most inhibition at a lower concentration (0.5%). Unlike KC70, *B. subtilis* SU40 maintained high activity even in the presence of SDS and Triton X-100, indicating a more robust enzyme stabilization mechanism [50]. The differences observed between KC70 and *B. subtilis* SU40 suggest variations in enzyme folding, membrane composition, or stress response mechanisms that influence surfactant tolerance. These findings reinforce the importance of strain-specific optimization when selecting microbial candidates for industrial enzyme production. This suggests that ionic surfactants disrupt the enzyme’s structural integrity, likely by interfering with hydrophobic interactions crucial for maintaining enzyme stability. The positive effect of Tween 80, a non-ionic surfactant, is consistent with previous findings indicating that non-ionic surfactants can help prevent enzyme aggregation and enhance solubility [68]. In the rumen environment, oxidative stress can be triggered by rapid microbial fermentation, dietary shifts, and metabolic overload, leading to reactive oxygen species (ROS) generation that challenges microbial survival [71,72]. Therefore, KC94’s demonstrated tolerance to oxidative stress may offer a colonization advantage in this highly competitive ecosystem. Few rumen studies have directly linked cellulase activity to oxidative/surfactant tolerance. KC94’s performance fills this gap and suggests a unique adaptation that could be exploited for industrial enzyme cocktails requiring stability under pretreated biomass conditions.

### 4.4. Diversity of Possible Cellulase and Hemicellulase Gene Fragments in KC94

The successful amplification of GH39, GH45, and GH48 genes indicates that KC94 possesses a strong genetic basis for lignocellulose degradation. While the expected fragments were confirmed in agreement with published data [40], the presence of additional PCR products suggests that the KC94 strain may contain multiple isoforms or gene variants of β-xylosidase and cellobiohydrolase. This observation is consistent with the genetic diversity often reported in cellulolytic microbial systems [73] and may indicate that KC94 possesses a broader enzymatic diversity for lignocellulose degradation than initially anticipated. One possible explanation for these additional products is the presence of paralogous genes, reflecting evolutionary adaptation of KC94 to efficiently hydrolyze diverse plant polysaccharides. Another explanation could be primer slippage due to the degeneracy of primers, as previously noted by Sheng et al. (2015) [40]. Nevertheless, the detection of multiple amplicons is encouraging, as it points toward functional redundancy or specialization within KC94, potentially enhancing its lignocellulolytic efficiency, which is characteristic of the rumen environment. Future sequencing of these amplicons will clarify whether these represent novel gene variants or artifacts of amplification, but the current findings already highlight KC94 as a promising source of diverse cellulolytic enzymes.

## 5. Conclusions

The cellulase enzymes produced by *Bacillus* spp. (KC50 and KC70) and *P. mirabilis* KC94 exhibit robust activity, pH and temperature stability, and tolerance to mild oxidative stress, making them suitable candidates for industrial applications in lignocellulosic biomass processing. The molecular identification of *P. mirabilis* KC94 as a cellulase producer adds to the diversity of microorganisms that can be utilized for lignocellulose degradation. This strain may complement traditional cellulolytic bacteria, contributing enzymes that function under diverse environmental and process conditions. Incorporating a broader diversity of bacterial strains could improve pretreatment efficiency, enzyme stability, and overall bioconversion performance in biofuel or biochemical production systems.

The cellulases produced by *P. mirabilis* KC94 have several features that could support integration into industrial bioenergy systems. Its enzyme activity at neutral pH and 35 °C, combined with tolerance to mild oxidative stress, makes KC94 a promising candidate for pretreatment of lignocellulosic biomass under moderate conditions. However, as KC94 exhibits sensitivity to surfactants such as SDS and Triton X-100, its application may require formulation strategies or co-culture systems to enhance enzyme stability and performance in industrial settings. The amplification of GH39, GH45, and GH48 genes confirms that KC94 harbors key cellulolytic genes and potentially multiple isoforms, reflecting genetic diversity that may enhance lignocellulose degradation. Such diversity is valuable for industry, where enzyme robustness and redundancy can improve efficiency in biomass conversion. Moreover, these findings complement lignin-degrading enzyme systems, supporting the design of co-culture strategies that integrate cellulose and lignin degraders for more effective bioprocessing. Although further sequencing of the amplicons is still needed to validate the variants, KC94 already stands out as a promising candidate for biotechnological applications. Integrating KC94 cellulases with more robust *Bacillus* spp. could provide complementary enzymatic activities, potentially improving overall hydrolysis efficiency. Future work will focus on evaluating enzyme yield at scale, co-culture synergy, tolerance to industrial stressors, and pretreatment of various lignocellulosic biomass, as well as evaluating apparent Km and Vmax for the most promising isolates (KC50, KC70, KC94) under their identified optimal conditions, thereby determining the feasibility of deploying them in sustainable bioenergy production workflows.

## Figures and Tables

**Table 1:** Comparison of cellulolytic potential among isolates based on clear zone diameter. Error bars represent ±SD; *n* = 3. Strain KC94 was significantly higher than KC50 and KC70 across all the plates (Tukey’s HSD, *p* < 0.001).

Isolate	Colony Diameter (mm)	Clear Zone Diameter (mm)	Relative Enzyme Activity
KC40	7.33 ± 0.12	16.21 ± 0.12	2.21 ± 0.03
KC50	62.16 ± 0.12	78.62 ± 0.11	1.26 ± 0.01
KC70	8.81 ± 0.10	23.41 ± 0.10	2.66 ± 0.02
KC94	5.99 ± 0.10	24.86 ± 0.15	4.15 ± 0.05

## Data Availability

The original contributions presented in this study are included in the article/Supplementary Material. Further inquiries can be directed to the corresponding author.

## Supplementary Materials

The following supporting information can be downloaded at https://www.mdpi.com/article/10.3390/microorganisms13092170/s1. Table S1 shows the cellulase production media that were used. Figure S1 shows the qualitative screening assay showing cellulase activity on 0.5% CMC agar plates after flooding with Congo Red dye. Table S2 includes the nucleotide accession numbers submitted to GenBank. Table S3: Relative abundance (number of isolates) of bacteria identified in this study. Table S4 covers the touchdown PCR cycling parameters for amplification of GH39, GH45, and GH48 gene fragments, as well as degenerate primers used to amplify cellulase and hemicellulase gene fragments. Statistical analysis for all the statistics in the manuscript is presented per factor per strain in the Excel sheets. References [40,74,75] are cited in the Supplementary Materials.

## References

[1] Ajala E.O., Ighalo J.O., Ajala M.A., Adeniyi A.G., Ayanshola A.M. (2021). Sugarcane bagasse: A biomass sufficiently applied for improving global energy, environment and economic sustainability. Bioresour. Bioprocess..

[2] Pan S., Zabed H.M., Wei Y., Qi X. (2022). Technoeconomic and environmental perspectives of biofuel production from sugarcane bagasse: Current status, challenges and future outlook. Ind. Crop. Prod..

[3] Alokika, Anu, Kumar A., Kumar V., Singh B. (2021). Cellulosic and hemicellulosic fractions of sugarcane bagasse: Potential, challenges and future perspective. Int. J. Biol. Macromol..

[4] Negrão D.R., Grandis A., Buckeridge M.S., Rocha G.J., Leal M.R.L., Driemeier C. (2021). Inorganics in sugarcane bagasse and straw and their impacts for bioenergy and biorefining: A review. Renew. Sustain. Energy Rev..

[5] Konde K.S., Nagarajan S., Kumar V., Patil S.V., Ranade V.V. (2021). Sugarcane bagasse based biorefineries in India: Potential and challenges. Sustain. Energy Fuels.

[6] Sarker T.C., Azam S.M.G.G., Bonanomi G. (2016). Recent advances in sugarcane industry solid by-products valorization. Waste Biomass Valorization.

[7] Liang B., Liang Y., Chen J., Zhu Y., Fu F., Gan T., Huang Z., Hu H., Zhang Y. (2024). Stepwise processing strategy for efficient and comprehensive utilization of sugarcane bagasse via enzymatic hydrolysis and catalytic conversion to produce 5-hydroxymethylfurfural. Ind. Crop. Prod..

[8] Kim Y.-K., Lee S.-C., Cho Y.-Y., Oh H.-J., Ko Y.H. (2012). Isolation of cellulolytic *Bacillus subtilis* strains from agricultural environments. ISRN Microbiol..

[9] Yin L.-J., Lin H.-H., Xiao Z.-R. (2010). Purification and characterization of a cellulase from *Bacillus subtilis* YJ1. J. Mar. Sci. Technol..

[10] Mushtaq Q., Ishtiaq U., Joly N., Qazi J.I., Martin P. (2024). Amylase and cellulase production from newly isolated *Bacillus subtilis* using acid treated potato peel waste. Microorganisms.

[11] Bhatt B., Bhatt K., Lal S., Srinivasan R., Bhatt V. (2024). Production of a novel cellulase by Bacillus amyloliquefaciens OKB3 isolated from soil: Purification and characterization. Int. J. Biol. Macromol..

[12] Liao Y., Wu S., Zhou G., Mei S., Yang Z., Li S., Jin Z., Deng Y., Wen M., Yang Y. (2024). Cellulolytic *Bacillus cereus* produces a variety of short-chain fatty acids and has potential as a probiotic. Microbiol. Spectr..

[13] Pană A.-M., Ordodi V., Gherman V., Sfîrloagă P., Dumitrel G.-A. (2023). Study on the Biodegradation Process of D-Mannose Glycopolymers in Liquid Media and Soil. Polymers.

[14] Araki Y., Mukaisho K., Sugihara H., Fujiyama Y., Hattori T. (2009). *Proteus mirabilis* sp. intestinal microflora grow in a dextran sulfate sodium-rich environment. Int. J. Mol. Med..

[15] Mahapatra S., Vickram A.S., Sridharan T.B., Parameswari R., Pathy M.R. (2016). Screening, production, optimization and characterization of β-glucosidase using microbes from shellfish waste. 3 Biotech.

[16] Patel D.D., Patel A.K., Parmar N.R., Shah T.M., Patel J.B., Pandya P.R., Joshi C.G. (2014). Microbial and Carbohydrate Active Enzyme profile of buffalo rumen metagenome and their alteration in response to variation in the diet. Gene.

[17] Patel V., Patel A.K., Parmar N.R., Patel A.B., Reddy B., Joshi C.G. (2014). Characterization of the rumen microbiome of Indian Kankrej cattle (*Bos indicus*) adapted to different forage diet. Appl. Microbiol. Biotechnol..

[18] Rabapane K.J., Matambo T.S. (2024). Profiling the dynamic adaptations of CAZyme-Producing microorganisms in the gastrointestinal tract of South African goats. Heliyon.

[19] Sarsan S., Merugu R. (2019). Role of bioprocess parameters to improve cellulase production: Part II. New and Future Developments in Microbial Biotechnology and Bioengineering.

[20] Kumari S., Sharma U., Krishna R., Sinha K. (2019). Screening and molecular characterization of cellulase producing actinobacteria from Litchi Orchard. Curr. Chem. Biol..

[21] Gaur R., Tiwari S. (2015). Isolation, production, purification and characterization of an organic-solvent-thermostable alkalophilic cellulase from *Bacillus vallismortis* RG-07. BMC Biotechnol..

[22] Hussain A.A., Abdel-Salam M.S., Abo-Ghalia H.H., Hegazy W.K., Hafez S.S. (2017). Optimization and molecular identification of novel cellulose degrading bacteria isolated from Egyptian environment. J. Genet. Eng. Biotechnol..

[23] Irfan M., Mushtaq Q., Tabssum F., Shakir H.A., Qazi J.I. (2017). Carboxymethyl cellulase production optimization from newly isolated thermophilic *Bacillus subtilis* K-18 for saccharification using response surface methodology. AMB Express.

[24] Hsiao N.-W., Chen Y., Kuan Y.-C., Lee Y.-C., Lee S.-K., Chan H.-H., Kao C.-H. (2014). Purification and characterization of an aspartic protease from the Rhizopus oryzae protease extract, Peptidase R. Electron. J. Biotechnol..

[25] Usman A., Mohammed S., Mamo J., Muleta D. (2021). Production, Optimization, and Characterization of an Acid Protease from a Filamentous Fungus by Solid-State Fermentation. Int. J. Microbiol..

[26] Weisburg W.G., Barns S.M., Pelletier D.A., Lane D.J. (1991). 16S ribosomal DNA amplification for phylogenetic study. J. Bacteriol..

[27] Wilson K.H., Blitchington R.B., Greene R.C. (1990). Amplification of bacterial 16S ribosomal DNA with polymerase chain reaction. J. Clin. Microbiol..

[28] Irfan M., Tayyab A., Hasan F., Khan S., Badshah M., Shah A.A. (2017). Production and characterization of organic solvent-tolerant cellulase from Bacillus amyloliquefaciens AK9 isolated from hot spring. Appl. Biochem. Biotechnol..

[29] Hall T.A. (1999). BioEdit: A User-Friendly Biological Sequence Alignment Editor and Analysis Program for Windows 95/98/NT.

[30] Waheeb M.S., Elkhatib W.F., Yassien M.A., Hassouna N.A. (2024). Optimized production and characterization of a thermostable cellulase from *Streptomyces thermodiastaticus* strain. AMB Express.

[31] Potprommanee L., Wang X.-Q., Han Y.-J., Nyobe D., Peng Y.-P., Huang Q., Liu J.-Y., Liao Y.-L., Chang K.-L., Yang S. (2017). Characterization of a thermophilic cellulase from *Geobacillus* sp. HTA426, an efficient cellulase-producer on alkali pretreated of lignocellulosic biomass. PLoS ONE.

[32] Nkuna R., Matambo T.S. (2024). Determining the metabolic processes of metal-tolerant fungi isolated from mine tailings for bioleaching. Minerals.

[33] Bernfeld P. (1955). Amylases, alpha and beta. Methods Enzymol..

[34] Alamri S.A., Mostafa Y., Alrumman S.A. (2016). Isolation, Screening and Optimization of *Geobacillus* stearothermophilus Cellulase Production using Date Palm Cellulosic Wastes. J. Pure Appl. Microbiol..

[35] Demissie M.S., Legesse N.H., Tesema A.A. (2024). Isolation and characterization of cellulase producing bacteria from forest, cow dung, Dashen brewery and agro-industrial waste. PLoS ONE.

[36] Gebbie L., Dam T.T., Ainscough R., Palfreyman R., Cao L., Harrison M., O’hAra I., Speight R. (2020). A snapshot of microbial diversity and function in an undisturbed sugarcane bagasse pile. BMC Biotechnol..

[37] Shareef I., Gopinath S.M., Satheesh M., Christopher S.X. (2015). Isolation and identification of cellulose degrading microbes. Int. J. Innov. Res. Sci. Eng. Technol..

[38] Singh S., Jaiswal D.K., Sivakumar N., Verma J.P. (2019). Developing efficient thermophilic cellulose degrading consortium for glucose production from different agro-residues. Front. Energy Res..

[39] Barman D., Dkhar M.S. (2022). Characterization and purification of esterase from Cellulomonas fimi DB19 isolated from Zanthoxylum armatum with its possible role in diesel biodegradation. Arch. Microbiol..

[40] Sheng P., Li Y., Marshall S.D.G., Zhang H. (2015). High genetic diversity of microbial cellulase and hemicellulase genes in the hindgut of Holotrichia parallela larvae. Int. J. Mol. Sci..

[41] Islam M., Sarkar P.K., Suzauddula M., Mohiuddin A. (2019). Optimization of fermentation condition for cellulase enzyme production from Bacillus sp. Malays. J. Halal Res..

[42] El-Khamisi E.F., Soliman E.A.M., El-Sayed G.M., Nour S.A., Abdel-Monem M.O., Hassan M.G. (2024). Optimization, gene cloning, expression, and molecular docking insights for enhanced cellulase enzyme production by *Bacillus amyloliquefaciens* strain elh1. Microb. Cell Factories.

[43] Malik W.A., Khan H.M., Javed S. (2021). Bioprocess optimization for enhanced production of bacterial cellulase and hydrolysis of sugarcane bagasse. BioEnergy Res..

[44] Mobley H.L.T., Pearson M. (2019). *Proteus mirabilis* overview. Proteus Mirabilis: Methods and Protocols.

[45] Patakova P., Kolek J., Sedlar K., Koscova P., Branska B., Kupkova K., Paulova L., Provaznik I. (2018). Comparative analysis of high butanol tolerance and production in clostridia. Biotechnol. Adv..

[46] Russmayer H., Marx H., Sauer M. (2019). Microbial 2-butanol production with *Lactobacillus diolivorans*. Biotechnol. Biofuels.

[47] Zaks A., Klibanov A.M. (1988). Enzymatic catalysis in nonaqueous solvents. J. Biol. Chem..

[48] Skovgaard P.A., Jørgensen H. (2013). Influence of high temperature and ethanol on thermostable lignocellulolytic enzymes. J. Ind. Microbiol. Biotechnol..

[49] Saini A., Aggarwal N.K., Yadav A. (2017). IIsolation and screening of cellulose hydrolyzing bacteria from different ecological niches. Bioeng. Biosci..

[50] Asha B.M., Sakthivel N. (2014). Production, purification and characterization of a new cellulase from *Bacillus subtilis* that exhibit halophilic, alkalophilic and solvent-tolerant properties. Ann. Microbiol..

[51] Rabapane K.J., Ijoma G.N., Matambo T.S. (2022). Insufficiency in functional genomics studies, data, and applications: A case study of bio-prospecting research in ruminant microbiome. Front. Genet..

[52] Zhang Q., Jian L., Yao D., Rao B., Xia Y., Hu K., Li S., Shen Y., Cao M., Qin A. (2023). The structural basis of the pH-homeostasis mediated by the Cl−/HCO3− exchanger, AE2. Nat. Commun..

[53] Wasfi R., Hamed S.M., Amer M.A., Fahmy L.I. (2020). *Proteus mirabilis* biofilm: Development and therapeutic strategies. Front. Cell. Infect. Microbiol..

[54] Chakkour M., Hammoud Z., Farhat S., El Roz A., Ezzeddine Z., Ghssein G. (2024). Overview of *Proteus mirabilis* pathogenicity and virulence. Insights into the role of metals. Front. Microbiol..

[55] Yang W., Beauchemin K. (2006). Physically effective fiber: Method of determination and effects on chewing, ruminal acidosis, and digestion by dairy cows. J. Dairy Sci..

[56] Hossain E. (2020). Sub-acute ruminal acidosis in dairy cows: Its causes, consequences and preventive measures. Online J. Anim. Feed. Res..

[57] Lian H., Zhang C., Liu Y., Li W., Fu T., Gao T., Zhang L. (2022). In Vitro Gene Expression Responses of Bovine Rumen Epithelial Cells to Different pH Stresses. Animals.

[58] Russell J.B., Wilson D.B. (1996). Why Are Ruminal Cellulolytic Bacteria Unable to Digest Cellulose at Low pH?. J. Dairy Sci..

[59] Hyder I., Reddy R.K., Raju J., Manjari P., Prasad S., Kumar A., Sejian V. (2017). Alteration in rumen functions and diet digestibility during heat stress in sheep. Sheep Production Adapting to Climate Change.

[60] Castro-Costa A., Salama A., Moll X., Aguiló J., Caja G. (2015). Using wireless rumen sensors for evaluating the effects of diet and ambient temperature in nonlactating dairy goats. J. Dairy Sci..

[61] Xu Z., Cen Y.-K., Zou S.-P., Xue Y.-P., Zheng Y.-G. (2019). Recent advances in the improvement of enzyme thermostability by structure modification. Crit. Rev. Biotechnol..

[62] Rahban M., Zolghadri S., Salehi N., Ahmad F., Haertlé T., Rezaei-Ghaleh N., Sawyer L., Saboury A.A. (2022). Thermal stability enhancement: Fundamental concepts of protein engineering strategies to manipulate the flexible structure. Int. J. Biol. Macromol..

[63] Wu H., Chen Q., Zhang W., Mu W. (2021). Overview of strategies for developing high thermostability industrial enzymes: Discovery, mechanism, modification and challenges. Crit. Rev. Food Sci. Nutr..

[64] Günel G., İnce O., Uzun Ö., Erdem E.I., İnce B. (2025). Enhancing biomethane production from cattle manure by integrating rumen bacteria: A microbial analyses with next-generation sequencing and quantitative PCR. Biomass Convers. Biorefinery.

[65] Oztat K., Yavuz A.A., Işçen C.F. (2024). Optimization studies on laccase activity of *Proteus mirabilis* isolated from treatment sludge of textile industry factories. Braz. J. Microbiol..

[66] Koike S., Kobayashi Y. (2009). Fibrolytic Rumen Bacteria: Their Ecology and Functions. Asian-Australasian J. Anim. Sci..

[67] Cheng B., Wu S., Liu S., Rodriguez-Aliaga P., Yu J., Cui S. (2015). Protein denaturation at a single-molecule level: The effect of nonpolar environments and its implications on the unfolding mechanism by proteases. Nanoscale.

[68] Thomas L., Ram H., Kumar A., Singh V.P. (2016). Production, Optimization, and Characterization of Organic Solvent Tolerant Cellulases from a Lignocellulosic Waste-Degrading Actinobacterium, *Promicromonospora* sp. VP111. Appl. Biochem. Biotechnol..

[69] Liu K., Zhang Y., Yu Z., Xu Q., Zheng N., Zhao S., Huang G., Wang J. (2021). Ruminal microbiota–host interaction and its effect on nutrient metabolism. Anim. Nutr..

[70] Silva É.B.R.d., Silva J.A.R.d., Silva W.C.d., Belo T.S., Sousa C.E.L., Santos M.R.P.d., Neves K.A.L., Rodrigues T.C.G.d.C., Camargo-Júnior R.N.C., Lourenço-Júnior J.d.B. (2024). A Review of the Rumen Microbiota and the Different Molecular Techniques Used to Identify Microorganisms Found in the Rumen Fluid of Ruminants. Animals.

[71] Celi P., Mandelker L., Vajdovich P. (2011). Oxidative stress in ruminants. Studies on Veterinary Medicine.

[72] Celi P., Chauhan S.S., Cottrell J.J., Dunshea F.R., Lean I.J., Leury B.J., Liu F. (2014). Oxidative stress in ruminants: Enhancing productivity through antioxidant supplementation. Feed. Broadening Horiz..

[73] Hua M., Yu S., Ma Y., Chen S., Li F. (2018). Genetic diversity detection and gene discovery of novel glycoside hydrolase family 48 from soil environmental genomic DNA. Ann. Microbiol..

[74] Singh K., Richa K., Bose H., Karthik L., Kumar G., Bhaskara Rao K.V. (2014). Statistical media optimization and cellulase production from marine Bacillus VITRKHB. 3 Biotech.

[75] Wei Z.-J., Zhou L.-C., Chen H., Chen G.-H. (2011). Optimization of the fermentation conditions for 1-deoxynojirimycin production by *Streptomyces lawendulae* applying the response surface methodology. Int. J. Food Eng..

