# Supplementary material for: Bioprospecting of Goat Rumen Microbiota for Optimum Cellulase Enzyme Production to Support Sustainable Bioenergy Systems
Source: Microorganisms. 2025 Sep 17;13(9):2170. doi: 10.3390/microorganisms13092170 (PMC12472465; doi:10.3390/microorganisms13092170)
Supplement: Supplementary file 1 [file microorganisms-13-02170-s001.zip › Supplementary Material Microorganisms-3837441.docx]

**Supplementary Material**

| **Name** | **Composition** | **Ref** |
| --- | --- | --- |
| Cellulase production media | (g/L) Carboxymethyl Cellulose (CMC) (10), peptone (10), NaCl (1), CaCl_2_ (0.005), MgSO_4_ (0.82), K_2_HPO_4_ (1.25), KH_2_PO_4_ (3), FeSO_4_ (0.01), ZnSO4 (0.005), MnCl_2_ (0.0001), and NH_4_Cl (1) | (Singh et al., 2014, Wei et al., 2011) |
| CMC Agar | Bacteriological agar (5 g/L), CMC (5 g/L) |  |

***Supplementary Table S1***: List of media Composition

***Supplementary Figure S1*:** Qualitative Assay showing cellulase activity on 0.5% CMC agar

| **Strain** | **Back** | **Front** |
| --- | --- | --- |
| **KC40** | 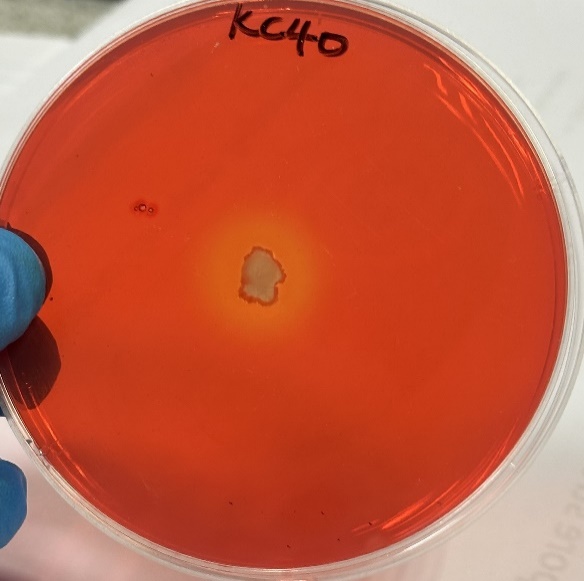 | 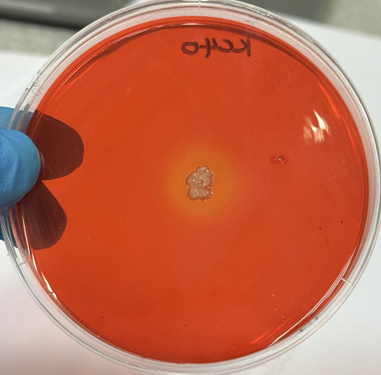 |
| **KC50** | 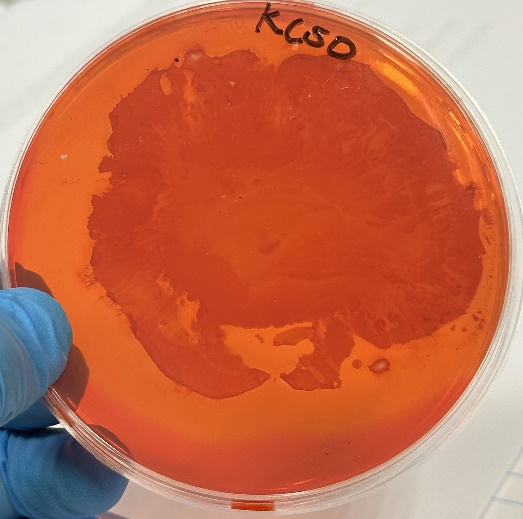 | 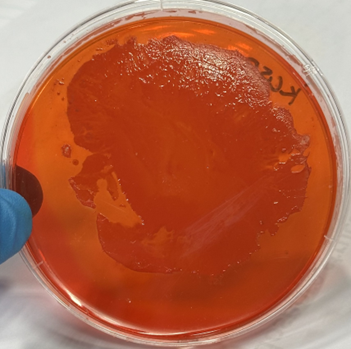 |
| **KC70** | 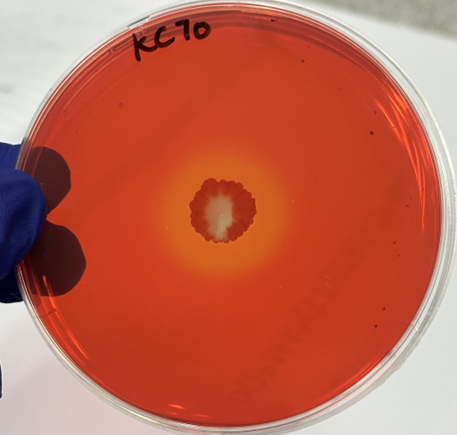 | 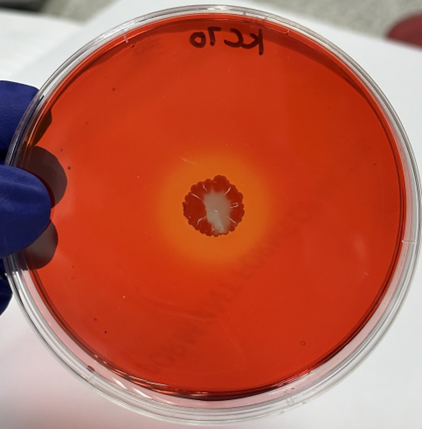 |
| **KC94** | 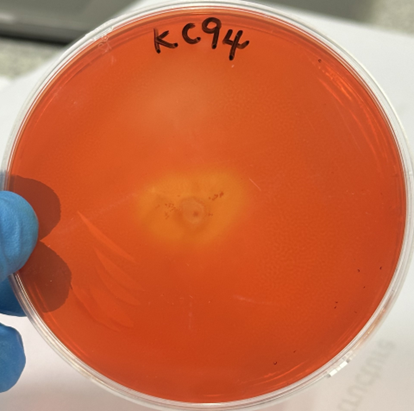 | 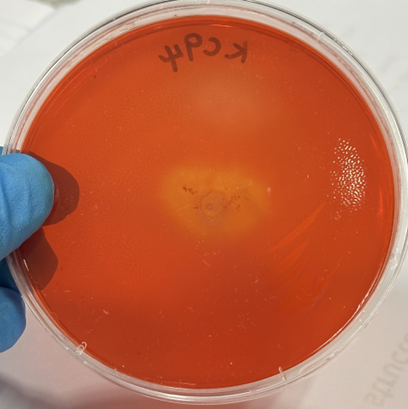 |

***Supplementary*  Table S2:** The nucleotide sequences generated in this study have been deposited in GenBank under the following accession numbers (under submission ID SUB15584114):

| **Sample ID** | **GenBank Accession No.** |
| --- | --- |
| KC40 | PX279117 |
| KC50 | PX279118 |
| KC70 | PX279119 |
| KC94 | PX279120 |

***Supplementary*  Table S3:** Operational taxonomic units of bacterial isolates clustered at 98% similarity. The identity of the representative isolates is presented at a genus level.

| **Representative (OTU)** | **OTUs (Accession Number and Sample ID)** | **Frequency** |
| --- | --- | --- |
| NR180419.1 | SJ02; LMN103; JCM; LMN103; KOD; KC70; OTG022; NR180419.1; ZHX3; SK2; YNB13; KC50; IS225; HBUAS64135; KC40; BFAM11; HBUAS69901; HBUAS73063; HBUAS67304; | 18 |
| D53 | swupm3; D53; beavul.ww1; S74-3-2; KC94; gut_R1_BHI_10-3_3A2; | 6 |

***Supplementary Table S4:*** Touchdown PCR cycling parameters for amplification of GH39, GH45, and GH48 gene fragments and degenerate primers used to amplify cellulase and hemicellulase gene fragments (Sheng et al., 2015).

|  |  | **1^st^ Stage** |  |
| --- | --- | --- | --- |
|  | 30 Cycles | Temperature | Time |
|  | Initial Denaturation  Denaturation  Annealing  Extension | 94 ^o^C  94 ^o^C  X ^o^C (-0.5 ^o^C/cycle)  72 ^o^C | 5 minutes  30s  30s  30s |
|  |  | **2^nd^ Stage** |  |
|  | 20 Cycles | Temperature | Time |
|  | Denaturation  Annealing  Extension  Final Extension  Cool down | 94 ^o^C  Y ^o^C  72 ^o^C  72 ^o^C  4 ^o^C | 30s  30s  30s  10 minutes  Infinity |
| **The following PCR settings are the same as the Standard** | | | |
| X = 65 Y = 50 | | | |
| X = 68 Y = 55 | | | |
| X = 65 Y = 50 | | | |
| **Pfam Family** | **Target Enzyme** | **Primer (5ʹ–3ʹ)** | **Length bp** |
| GH39 (PF01229) | β-Xylosidase | GH39F: TTYGARGTNTGGAAYGARCC  GH39R: GCRTGNCKISWIACRAARTC | 223–230 |
| GH45 (PF02015) | β-1,4-Endoglucanase | GH45F: ACCMGITAYTGGGAYTGYTG  GH45R: AAGRYICCNAVICCNCCICCNGG | 377–413 |
| GH48 (PF02011) | Cellobio-Hydrolase | GH48F: GARGCNCCNGAYYAYGGICA  GH48R: CCNCGYTGRWAIGT | 420 |

SHENG, P., LI, Y., MARSHALL, S. D. & ZHANG, H. 2015. High genetic diversity of microbial cellulase and hemicellulase genes in the hindgut of Holotrichia parallela larvae. *International Journal of Molecular Sciences,* 16**,** 16545-16559.

SINGH, K., RICHA, K., BOSE, H., KARTHIK, L., KUMAR, G. & BHASKARA RAO, K. V. 2014. Statistical media optimization and cellulase production from marine Bacillus VITRKHB. *3 Biotech,* 4**,** 591-598.

WEI, Z.-J., ZHOU, L.-C., CHEN, H. & CHEN, G.-H. 2011. Optimization of the fermentation conditions for 1-deoxynojirimycin production by Streptomyces lawendulae applying the response surface methodology. *International Journal of Food Engineering,* 7.

**References**
